# Supplementary figures and images for: Impact of screening and follow‐up colonoscopy adenoma sensitivity on colorectal cancer screening outcomes in the CRC‐AIM microsimulation model
Source: Cancer Med. 2020 Dec 13;10(8):2855–64. doi: 10.1002/cam4.3662 (PMC8026922; doi:10.1002/cam4.3662)

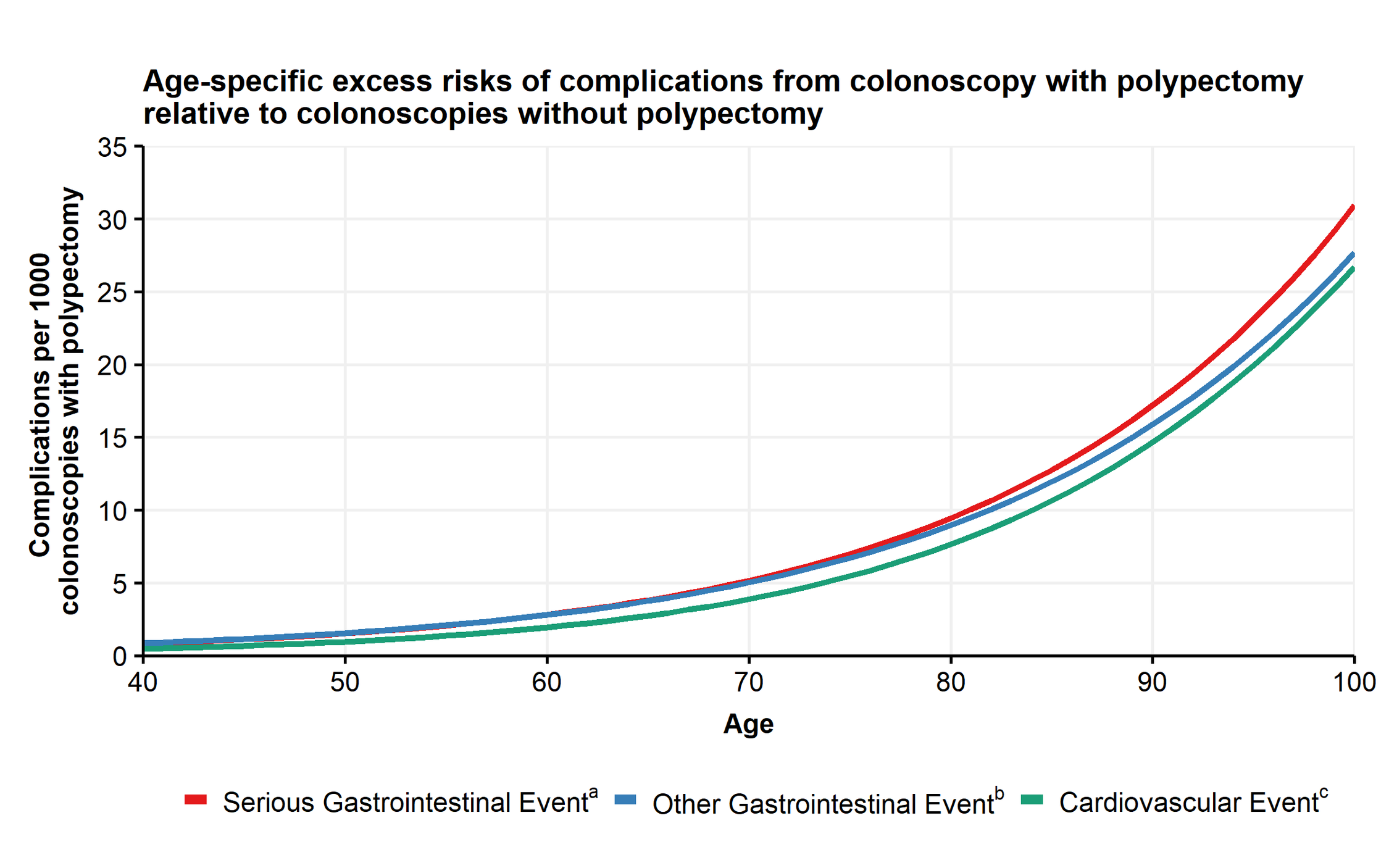

Supplement: Supplementary file 1 — Fig S1 [file CAM4-10-2855-s004.tif]

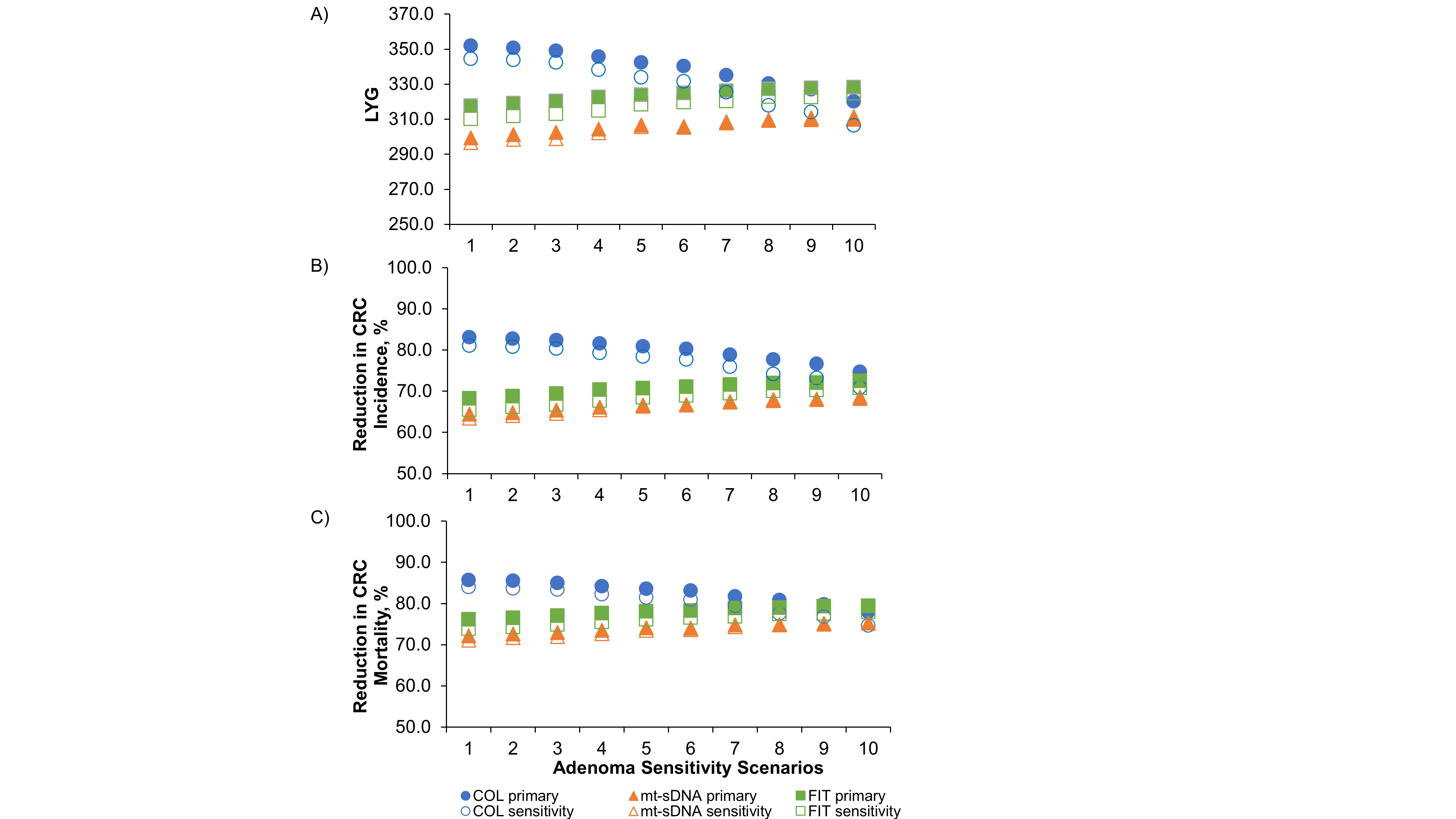

Supplement: Supplementary file 2 — Fig S2 [file CAM4-10-2855-s002.tif]

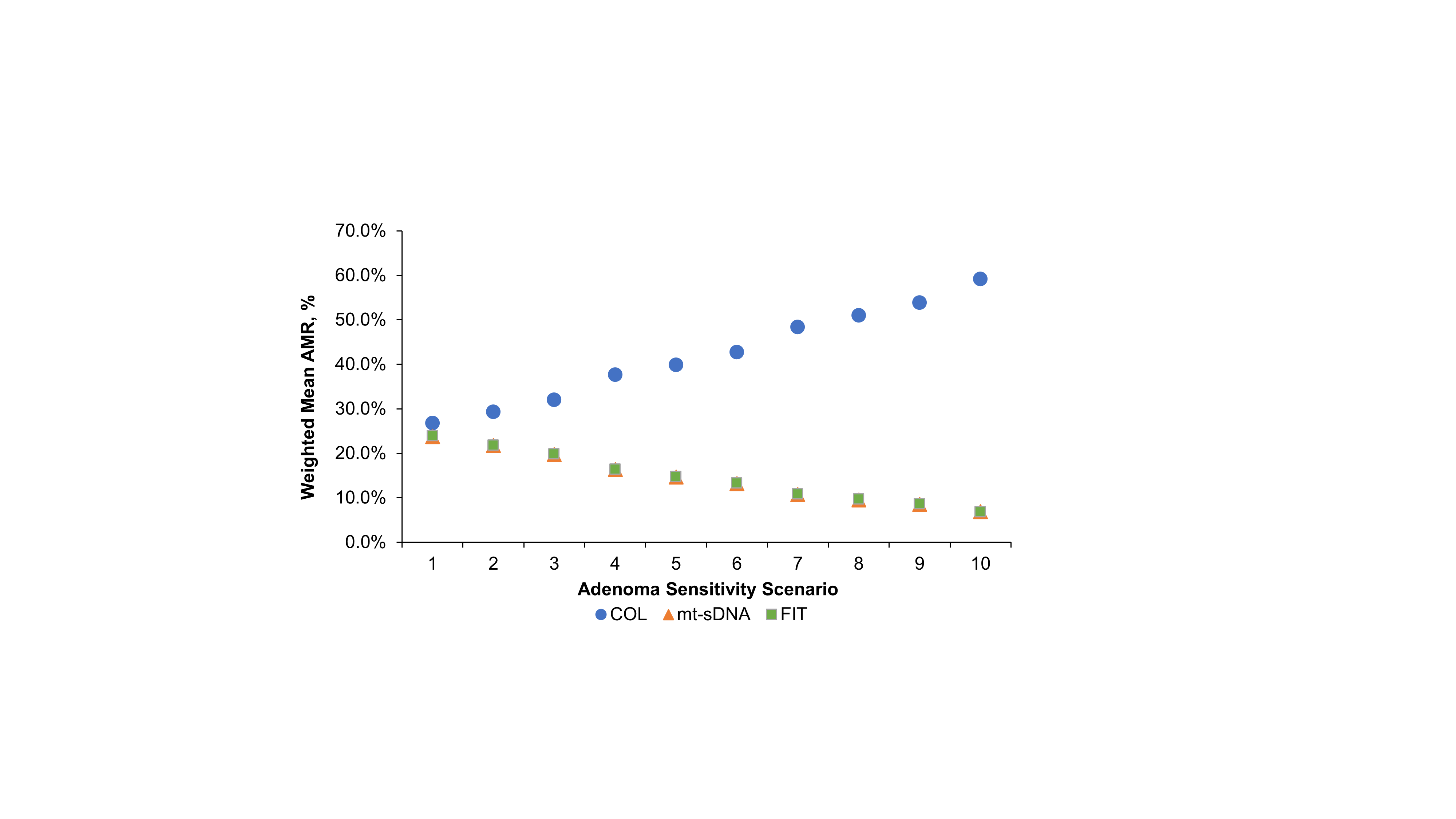

Supplement: Supplementary file 3 — Fig S3 [file CAM4-10-2855-s001.tif]
